# Perfectly Coupled Transcriptional Regulation of Fatty Acid Synthesis in Enterocci, Streptocci, and Lactococci

**DOI:** 10.1111/mmi.70054

**Published:** 2026-02-16

**Authors:** Qi Zou, John E. Cronan

**Affiliations:** ^1^ Department of Microbiology University of Illinois at Urbana‐Champaign Urbana Illinois USA; ^2^ Department of Biochemistry University of Illinois at Urbana‐Champaign Urbana Illinois USA

The fatty acid synthesis operon of *Enterococcus faecalis* and similar operons in related pathogenic bacteria are unusual in that *fabT*, the gene encoding the transcriptional repressor, is the first gene of the multigene fatty acid synthesis operon that it regulates (Figure 1). Therefore, the levels of FabT and the operon encoded enzymes are coordinated because they are encoded on the same mRNA. This is the case in *E. faecalis* (Bi et al. 2014; Hays et al. 2021; Zhu et al. 2019; Zou et al. 2022), the streptococci (e.g., *Streptococcus pneumoniae* ) (Jerga and Rock 2009; Yao and Rock 2015, 2017) (Lambert et al. 2022) and lactococci (Eckhardt et al. 2013). Moreover, the FabT regulatory ligands, acyl‐ACPs, which bind FabT to increase operator binding (Jerga and Rock 2009; Zhu et al. 2019; Zou et al. 2022; Lambert et al. 2023, 2022), are produced by genes regulated by FabT.

**FIGURE 1:**
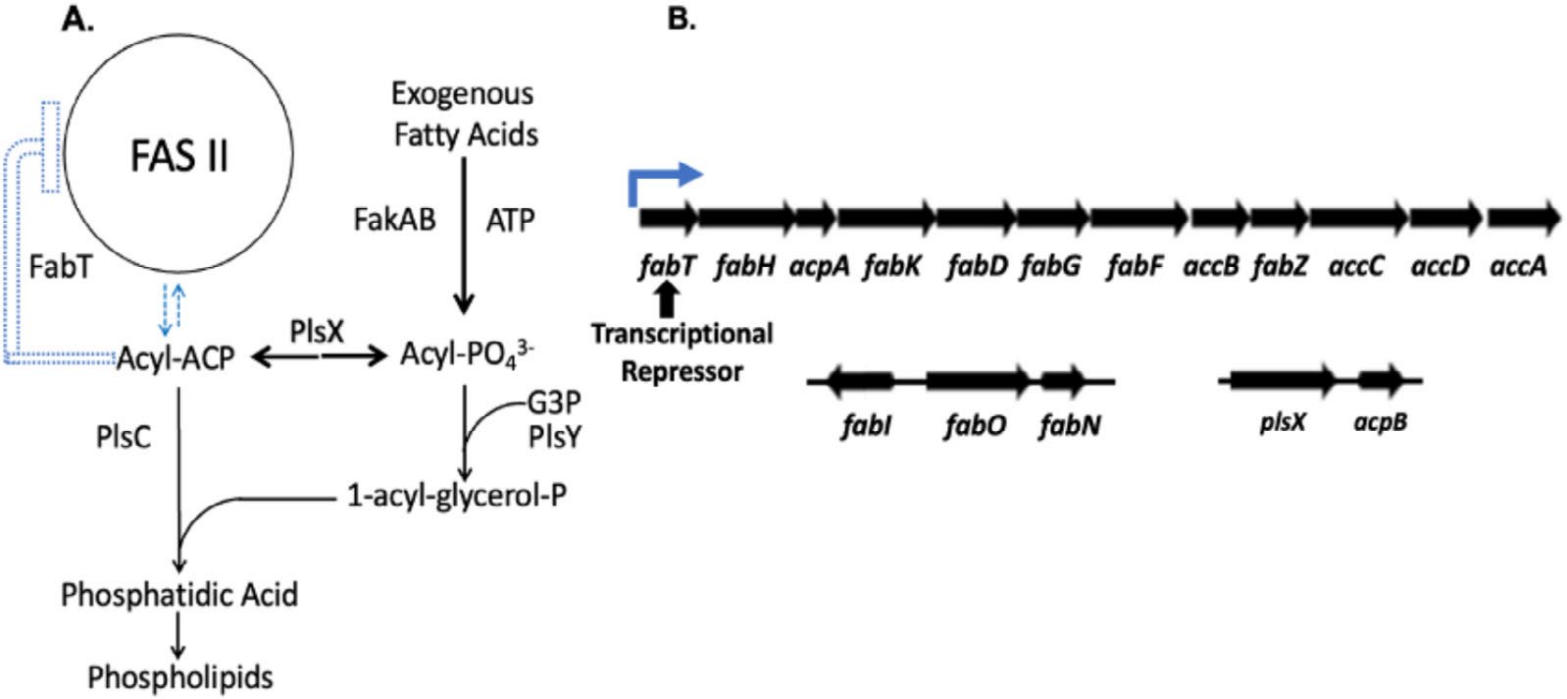
Fatty acid metabolism in the Lactobacillales and the *E. faecalis* fatty acid metabolism operons. (A) The pathways of de novo synthesis and of exogenous fatty acid incorporation. (B) The twelve gene *E. faecalis* FabT operon is transcribed from the blue promoter and transcription proceeds from upstream of *fabT* through *accA* (Bi et al. 2014; Hays et al. 2021). The operon *fabK* gene is cryptic (Bi et al. 2014). The *fabI, fabO*, and *fabN* genes are part of the FabT regulon whereas the *plsX*, *acpB*, and *fakAB* genes are not (Dong et al. 2025; Zou et al. 2022). AcpB functions in fatty acid incorporation and FabT repression but cannot replace AcpA (Dong and Cronan 2022; Zhu et al. 2019). FabT is a member of the MarR family of dimeric winged helix‐turn‐helix proteins (Deochand and Grove 2017; Zuo et al. 2019). Note that streptococcal operons have a FabT‐regulated promoter located between the *acpA* and *fabK* genes (Lu and Rock 2006) that is absent in *E. faecalis* (Bi et al. 2014; Hays et al. 2021).

The conservation of this operon arrangement in bacteria that occupy very different environmental niches argues that it plays an important role. This operon arrangement seems highly evolved in that the coding sequences of *fabT* and *fabH*, the gene encoding the first pathway enzyme, often minimally overlap or directly abut. Transcription of the FabT repressor gene and the genes responsible for synthesis of the regulatory ligands (acyl‐ACPs) are coupled. Each of the regulatory partners controls transcription of the other partner. FabT decreases transcription of the genes required for acyl‐ACP synthesis and acyl‐ACPs bind FabT and decrease FabT transcription by strengthening autoregulation. The fact that the FabT repressor is encoded on the same mRNA that encodes most of the fatty acid synthesis genes makes these operons examples of the perfectly coupled system of Hlavacek and Savageau (1997). In their extensive mathematical modeling of bacterial regulatory systems, they defined a perfectly coupled system in which the transcription of the regulatory protein is coordinated with the transcription of the regulated enzymes (Hlavacek and Savageau 1997). In complete uncoupling, the other extreme, there is a constant supply of the regulatory protein. Hlavacek and Savageau predict that in negatively regulated systems, such as the FabT operons, perfect coupling provides optimal regulatory performance (Hlavacek and Savageau 1997).

Another aspect of fatty acid synthesis conserved in these bacteria is that FabT repression is weak relative to other negatively regulated bacterial operons. Relative to the wild type strain, deletion of the *fabT* gene results in only a 2‐ to 5‐fold increase in operon transcription without effect on growth (Eckhardt et al. 2013; Jerga and Rock 2009; Lambert et al. 2023; Yao and Rock 2013; Zou et al. 2022; Hays et al. 2021; Faustoferri et al. 2015).

A reviewer queried whether weak repression is due to limiting production of FabT or to weak operator binding by the repressor. Electrophoretic gel shift analyses using purified FabT proteins indicate that operator binding is weak. *E. faecalis* FabT required a 20‐fold molar excess of FabT to give complete mobility shift of operator DNA (Zou et al. 2022) whereas in *S. pneumoniae* and *Lactococcus lactis* 25‐fold and 40‐fold molar excesses of FabT were required for complete operator gel shifting (Eckhardt et al. 2013; Lu and Rock 2006). Another gel shift analysis of *S. pneumoniae* FabT operator binding used 60 nm FabT and 10 pm promoter DNA (Jerga and Rock 2009). Comparison to a known strong repressor, TrpR of the *E. coli* tryptophan operon, illustrates the weakness of FabT operator binding. In the presence of tryptophan, TrpR repressor quantitatively shifts equimolar TrpO operator DNA fragments, a 1:1 ratio (Beckmann et al. 1993).

## 1 Regulation in the Presence or Absence of Exogenous Fatty Acids

The above bacterial species and other members of the order Lactobacillales avidly incorporate exogeneous fatty acids into their membrane phospholipids (Eckhardt et al. 2013; Lai and Cronan 2003; Lambert et al. 2023; Lu and Rock 2006; Morgan‐Kiss and Cronan 2008; Saito et al. 2018; Hays et al. 2021). Incorporation of de novo synthesized acyl chains into phospholipids is strongly outcompeted by exogeneous fatty acids due to increased FabT repression by acyl‐ACPs derived from the acids (see below). In *E. faecalis* and *S. pneumoniae* , exogenous oleic acid almost completely displaces the native C18 unsaturated species and becomes the dominant acyl chain (> 85%) of the membrane phospholipids (Zou et al. 2022; Dong et al. 2025) (Parsons et al. 2011). However, the phospholipids of oleate‐grown *E. faecalis* wild type cells contain saturated and C16 unsaturated acyl chains, indicating 10%–15% residual fatty acid synthesis. These low levels are due to FabT repression because deletion of FabT stimulates de novo fatty acid synthesis resulting in a large (9‐fold) decrease in oleic acid incorporation into phospholipids (Zou et al. 2022).

We propose that the residual fatty acid synthesis in wild type cells grown with exogenous fatty acids provides a buffer to ameliorate exhaustion of the fatty acid supply. The buffer would provide sufficient enzymes and acyl‐ACPs to ensure maintenance of a functional membrane phospholipid bilayer until de novo fatty acid synthesis can ramp up. This proposal predicts that strong FabT repression would block synthesis of the buffer and inhibit the transition from exogenous fatty acid utilization to full de novo fatty acid synthesis. Hence, the moderate nature of FabT repression seems a compromise. Weak FabT repression gives partial saving of cellular resources but allows sufficient residual expression to form a buffer.

**FIGURE 2:**
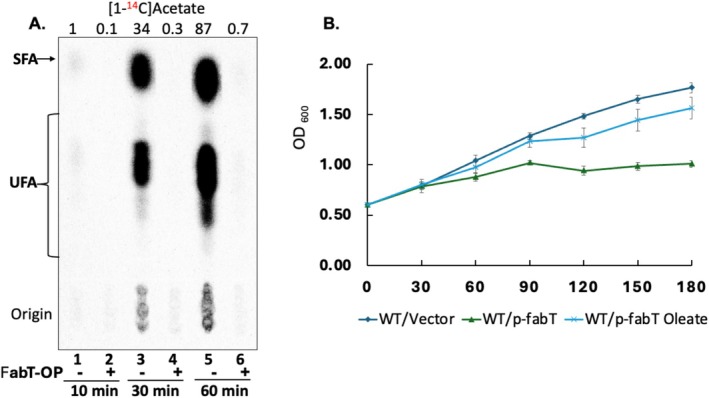
Recovery of de novo fatty acid synthesis following removal of oleic acid. Panel A shows incorporation of [1‐^14^C]acetate into phospholipid acyl chains in the wild type strain carrying the vector plasmid or the FabT overproduction plasmid, pFabT. Phosphorimaging of an argentation thin layer chromatography plate is shown (note the faint incorporations in lanes 1 and 6). The relative incorporation values are given at the top of the plate. Panel B shows growth of both strains in the absence of oleate and also that of the FabT overproduction strain in the presence of 100 μM oleate. The strains were grown with 100 μM oleate in M17 medium to mid log phase. Oleate was removed by pelleting the cells followed by several washes with 1xPBS (phosphate‐buffered saline) and resuspension in M17 medium. Note that FabT overproduction blocked [1‐^14^C]acetate incorporation at both 10 and 30 min after resuspension where growth of the strain parallels that of the wild type/vector strain. Note that after 60 min the [1‐^14^C]acetate incorporation of the FabT overproduction had not reached the level reached by the wild type/vector strain after 10 min. Radioactive labeling and lipid analyses were done as previously described (Zou et al. 2024, 2025, 2022).

We tested the buffer hypothesis by removing oleate from cultures of a wild type strain overproducing FabT (thereby mimicking a stronger repressor) and the wild type strain carrying the vector plasmid. Resumption of de novo fatty acid synthesis was assayed by [1‐^14^C]acetate incorporation into phospholipid acyl chains. The FabT overproduction strain was unable to restart de novo synthesis whereas the control strain promptly restarted synthesis (Figure 2).

A brief digression into fatty acid uptake in these bacteria is required because the pathway that converts exogenous acids into the acyl‐ACPs is not a direct activation to acyl‐CoA or acyl‐ACP species as in proteobacteria. These bacteria utilize the more complex FakAB pathway discovered by Rock and coworkers (Lu et al. 2006; Parsons et al. 2014) (for reviews see Yao and Rock 2015; Yao and Rock 2017; Cronan 2014). FakAB, a two‐component fatty acid kinase, activates incoming fatty acids by conversion to acyl‐phosphates. A portion of the acyl‐phosphate acyl chains is shuttled to ACP to form acyl‐ACPs in a freely reversible reaction catalyzed by PlsX (both acyl‐phosphates and acyl‐ACPs are required for phospholipid synthesis) (Yao and Rock 2015, 2017; Cronan 2014) (Figure 1). The resulting acyl‐ACPs bind FabT and decrease expression of both de novo fatty acid synthesis and FabT. Unusual in bacteria is that the enterococci and streptococci have a second ACP gene, *acpB*, that is not under FabT control (Zou et al. 2025, 2022; Lambert et al. 2023) (Figure 1). Acyl‐AcpB species function in exogenous fatty acid uptake but are also FabT regulatory ligands and acyl chain donors in phospholipid synthesis (Zhu et al. 2019; Dong and Cronan 2022). The ACP encoded within the FabT operon, AcpA, is the acyl carrier of fatty acid synthesis (Dong and Cronan 2022) and acyl‐AcpA species like acyl‐AcpB species function as FabT regulatory ligands and phospholipid acyl chain donors (Figure 1) (Lambert et al. 2023; Zou et al. 2025, 2022; Dong and Cronan 2022). Note that PlsX via acyl‐phosphate intermediates allows AcpA‐bound acyl chains to be transferred to AcpB and vice versa. Acyl‐AcpB species are significantly stronger FabT regulatory ligands than acyl‐AcpA species in *E. faecalis* (Zou et al. 2022) and are essentially the sole FabT repression ligand in *S. pyogenes* (Lambert et al. 2023). Like *E. faecalis* AcpB, expression of the other required fatty acid incorporation proteins (PlsX, FakA plus several FakBs) is independent of FabT (Dong et al. 2025; Lambert et al. 2023; Zou et al. 2022, 2025) and thus exogenous fatty acid incorporation is a constitutive pathway in these bacteria.

## 2 Overview

Does the regulation of fatty acid synthesis in *E. faecalis* and related bacteria differ from well‐understood repression pathways such as those of *E. coli* tryptophan and methionine synthesis? Based on the analysis of Hlavacek and Savageau (Hlavacek and Savageau 1997), this is the case. The FabT fatty acid synthesis operons are perfectly coupled whereas the above *E. coli* pathways have direct coupling, a form of coupling intermediate between perfect coupling and complete uncoupling (Hlavacek and Savageau 1997). Hlavacek and Savageau cite only one example of perfect coupling, the *E. coli* bifunctional alanyl‐tRNA synthetase, AlaS. AlaS catalyzes alanyl‐tRNA synthesis and negatively represses its own transcription dependent on alanine levels (Putney and Schmmel 1981). In the AlaS case perfect coupling comes from the properties of the protein whereas perfect coupling of the above Lactobacillales fatty acid synthesis operons results from cotranscription of the repressor and metabolic genes.

There seem few well‐studied examples other than the Lactobacillale *fabT* operons in which the repressor gene is cotranscribed with the genes it regulates. A reviewer suggested the Fe‐S cluster biogenesis operon, *iscRSUA*. However, although the *iscR* repressor gene is transcribed with the downstream genes, the small regulatory RNA, OxyS, binds the operon mRNA between the *iscR* and *iscS* coding sequences to decrease transcription of the downstream genes (Baussier et al. 2024). A second small regulatory RNA, FnrS, also uncouples *iscR* and *iscS* transcription by an unknown mechanism (Baussier et al. 2024). Hence, these small RNAs prevent perfect coupling of the *iscRSUA* operon despite cotranscription of the repressor and catalysis genes.

## Author Contributions

Q. Z. and J. E. C. wrote the original draft and the subsequent and final versions. Q. Z. performed the investigation. All authors have agreed to be responsible for ensuring the accuracy of the presented data.

## Data Availability

The data that support the findings of this study are available from the corresponding author upon reasonable request.
